# Clinical observation of posterior decompression, fusion and fixation in the treatment of spinal gout: a case series

**DOI:** 10.1186/s13018-023-03791-9

**Published:** 2023-04-15

**Authors:** M. M. Xingmao Zhou, M. M. Minhua Wu, M. M. Lili Sang, M. M. Junzhe Wu

**Affiliations:** 1 Department of Orthopaedic Surgery, Zhongshan Hospital of Traditional Chinese Medicine, Zhongshan City, 528400 Guangdong Province People’s Republic of China; 2 Division of Spine Surgery, Department of Orthopaedic Surgery, Zhongshan Hospital of Traditional Chinese Medicine, No. 3, Kangxin Road, West District, Zhongshan City, 528400 Guangdong Province People’s Republic of China

**Keywords:** Spinal gout, Posterior decompression, Fusion and fixation, Clinical effect

## Abstract

**Objective:**

The aim of the present study was to assess the effect of posterior decompression, fusion and fixation in the treatment of spinal gout. Spinal gout is a disease of gouty arthritis involving the spine, which can affect all segments of the spine. At present, the etiology and pathogenesis of spinal gout are not clear, and there are no definite methods for the treatment of spinal gout.

**Methods:**

This was a case series of seven patients (seven men) who underwent posterior decompression, fusion and fixation in the treatment of spinal gout between January 2016 and January 2020. Physical examination, radiography, CT, MRI, Japanese Orthopaedic Association (JOA) score and visual analog scale (VAS) score were used to evaluate the effect of this procedure. All patients were followed up every 3 months. The evaluation time point was 12 months after the operation. Comparisons of the functional indexes of the patients before and after the operation were performed using SPSS 22.0 (IBM, Armonk, NY, USA).

**Results:**

The JOA score was 13.43 ± 6.55 and the VAS score was 7.43 ± 1.51 preoperatively. The JOA score was 24.43 ± 3.74 and the VAS score was 0.86 ± 0.90 postoperatively at 12 months after surgery. At 12 months after surgery, the JOA and VAS score showed significant improvements when compared with those before surgery (*P* = 0.004 and *P* = 0.002, respectively). None of the patients had re-surgery of the gout due to actively and reasonably controlling uric acid. No loosening or displacement of screws was reported. There was only one screw tail cap loosening. Radiographic examination revealed that there was no obvious accumulation of gout or surrounding bone destruction, and the segmental instability was significantly improved. There was no progressive aggravation of neurological symptoms of the seven patients.

**Conclusions:**

Posterior approach decompression, fusion and fixation can stabilize the vertebral body, remove gout and directly relieve local spinal cord compression. The method is a reliable surgical choice for the treatment of spinal gout.

## Introduction

Spinal gout is a disease of gouty arthritis involving the spine, which can affect all segments of the spine. It is common in patients with long-term gout or hyperuricemia. Clinically, it can have symptoms of back pain, especially nerve compression symptoms such as limb numbness and arthralgia, and even paralysis [1]^.^ In 1950, Kersley reported a complex case with hyperuricemia and softening and subluxation of the first cervical vertebra and splenomegaly [2]. Vinstein AL showed narrowing of the intervertebral disk space at C3–4 and erosive changes in the end-plate and odontoid process due to gout [3]. Steven K. Magid MD demonstrated the spinal cord compression of a 50-year-old postmenopausal woman by tophi [4]. Spinal gout used to be recognized as a rare manifestation of gout, but recently, accruing cases have been reported. De Mello et al. demonstrated a high prevalence of axial gout not associated with spine symptoms with a total of forty-two patients with gout [5]. Toprover et al. reviewed 131 previously reported cases of spinal involvement in gout and conclude that spinal gout may be common and an underappreciated source of axial pain [6]. Ding reported a rare case of tophaceous gout in thoracic spine and reviewed a large number of vertebral gout in order and brought forth conclusions that gout can be involved in any spine level and the probability of occurrence of thoracic spine gout is 17.8% while the proportion of lumbar vertebrae and cervical vertebrae were 38% and 24.8%, respectively. Gout happens most in the cervical spine is C4, in the thoracic spine is T9 and in the lumbar vertebra is L5 [7].

At present, the etiology and pathogenesis of spinal gout are not clear, and there are no definite methods for the treatment of spinal gout. This paper retrospectively analyzed the clinical data of seven patients with spinal gout treated by posterior spinal decompression, fusion and fixation in zhongshan hospital of traditional Chinese medicine from January 2016 to January 2020, evaluated the efficacy of posterior spinal decompression, fusion and fixation in the treatment of 7 cases of spinal gout, and provided ideas for the selection of surgical methods for the treatment of spinal gout.

## Methods

### Diagnostic criteria, inclusion criteria, and exclusion criteria

#### Diagnostic criteria

The diagnosis of spinal gout was made by combining the positive serum uric acid. The physical examination, radiography, CT and MRI could be used to confirm the diagnosis. The tissue during the operation was taken out for pathological examination, and the result was indicating gout.

#### Inclusion criteria

The inclusion criteria were: (1) patients 18 to 75 years old who were diagnosed with spinal gout; (2) patients who underwent posterior decompression, fusion and fixation; and (3) all the follow-up data for serum uric acid, physical examination, radiography, CT, MRI, Japanese Orthopaedic Association (JOA) score and visual analog scale (VAS) score had been collected.

#### Exclusion criteria

The exclusion criteria were: (1) patients who disagreed with the surgical plan, were unwilling to participate in experimental research, or did not cooperate with the treatment; (2) patients with complications such as serious cardiovascular and cerebrovascular diseases, and liver, kidney or hematopoietic system diseases; (3) patients with fever or skin allergies, with mental illness or Alzheimer’s disease.

#### General data

From January 2016 to January 2020, seven patients diagnosed with spinal gout by serum uric acid, physical examination, radiography, CT, MRI were admitted to our hospital. The patients were all men with a mean age of 52.57 ± 20.98 years. The medical history of the patients was range from 4 days to 2 years. Follow-up was performed in all seven patients. The mean follow-up was 3.14 ± 9.4 months (range from 2 to 5 years). The suffered segments were C1, C2, L3, L4, L5 and S1 (Table 1).

**Table 1:** Clinical characters and spinal compression of the patients with tophaceous gout of the spine

Case	Age	Sex	HT of Gout (Y/N)	Serum uric acid (umol/L)	HT of symptom	Follow-up period	Clinical features	Neurological presentation	Involved vertebral level	Compression position	Treatment
1	26	M	Y	670	6 months	2 years	Numbness of both buttocks, radiation pain of both lower limbs	Bilateral dorsalis extensor and plantar flexor strength (4/5), perianal numbness	L4–S1	Erosion over adjacent vertebral bodies, facet articulation and lamina attachments, posterior spinal cord and nerve roots	Surgery of posterior decompression, L3–S1 fusion and fixation
2	74	M	N	/	2 years	5 years	Back pain, followed by both lower limbs weakness and radiation pain	Lower legs weakness (2/5), absent knee jerk reflexes, weakness of testicular reflex, anal reflex and sellar sensation	L4/5	L4/5 facet articulation, posterior spinal cord and nerve roots	Surgery of posterior decompression, L2–L5 fusion and fixation
3	76	M	Y	518	1 month	3 years	Back pain, followed by both lower limbs radiation pain	NO	L5/S1	Spinal cord	Surgery of posterior decompression, L5/S1 fusion and fixation
4	23	M	Y	562	4 days	3 years	Neck occipital pain	NO	C1/2	Atlantoaxial pathological fracture	Posterior atlantoaxial iliac bone graft fusion and internal fixation
5	65	M	Y	542	2 months	4 years	Back pain, followed by both lower limbs weakness and radiation pain	Left dorsalis extensor strength (4/5), weakness of testicular reflex, anal reflex and sellar sensation	L4/5	Erosion over adjacent vertebral bodies, facet articulation and lamina attachments, posterior spinal cord and nerve roots	Surgery of posterior decompression, L4–S1 fusion and fixation
6	39	M	Y	558	1 month	2 years	Back pain, followed by both lower limbs radiation pain	Bilateral lower limbs numbness, maximum temperature: 39.8^◦^C	L4–S1	Erosion over L4–S1 left adjacent vertebral bodies, facet articulation and lamina attachments, posterior spinal cord and nerve roots	Surgery of posterior decompression, L4–S1 fusion and fixation
7	65	M	N	/	10 days	3 years	Back pain, followed by both lower limbs radiation pain	Decreased skin sensation of right lower limb	L3–L5	Gout stones scattered between L3/4 and L4/5 interspinous ligaments, ligamentum flavum and annulus fibrosus, posterior spinal cord	Surgery of posterior decompression, L3–L5 fusion and fixation

General data of the patients are shown in Table 1. M: male; HT: history; Y: yes; N: no

### Treatment

All patients were prone position and underwent general anesthesia. Make a posterior median incision centered on the affected vertebra, peel off the fascia and muscles on both sides, and expose the vertebra. We should insert the screws, place the titanium rod for connection and apply the tail cap. And then we opened the posterior lamina of the spinal canal for decompression, and remove the gout in the spinal canal. At the same time, after the treatment of intervertebral space, an appropriate amount of autologous bone and interbody fusion cage or rear bone graft should be implanted. For the cases with gout invasion of vertebral lamina, pedicle and vertebral body, the whole vertebral lamina decompression should be adopted to completely remove the gout. A large number of gout had a clear boundary with the surrounding muscle tissue. It could be seen that the gout capsule exists, but the boundary with bone tissue was unclear, which may erode the vertebral lamina, vertebral body and spinal facet joint capsule. The intervertebral disc was mostly well preserved. Gout usually enters the spinal canal and adheres to the spinal cord and cauda equina. After separation and decompression, it was implanted with pedicle screw rod system for fixation. At the same time, an appropriate amount of autologous bone and intervertebral fusion cage were implanted after intervertebral space treatment. Wash the incision and suture the wound layer by layer. The tissue was sent for pathological examination.

### Rehabilitation after surgery

The patients should wear elastic waist or neck support for fixed braking for 4–6 weeks after operation. The patients could be guided to exercise muscle function 2 days after operation, and get out of bed 3 days after operation with the elastic waist or neck support.

### Follow-up and evaluation

All patients were followed up every 3 months after operation. The follow-up included the improvement of pain and nerve root symptoms, the evaluation of the improvement of patients' functional disorders, and the imaging evaluation of X-ray films and CT. The evaluation time point was 12 months after the operation.

### Outcome measures

#### Imaging evaluation

The anteroposterior and lateral X-ray films of the spinal segment where the lesion was located were obtained post-operation. The position of internal fixation and the effect of spinal fusion and fixation were observed. CT was used to observe and evaluated the improvement of spinal cord compression, the occurrence of new spine gout compared with preoperative imaging, the internal fixation device stability and the bone growth and healing of the surgical vertebral body.

#### JOA score

The JOA score and the VAS score were used to evaluate the effect of this procedure before and 12 months after the operation, which is effective at measuring changes in patients’ conditions. The JOA is a disease-specific, physician-oriented scale designed to assess the neurological status of a patient and allows surgeons to measure pre- and post-intervention changes. The scale involves a number of constructs including scoring of feeding, upper extremity shoulder and elbow function, lower extremity gait capabilities, sensory involvements, and bowel and bladder control [8, 9]. The highest JOA score of lumbar spine was 29 points. The highest JOA score of cervical spine was 17 points. The lowest was 0. The lower the score, the more obvious the dysfunction was.

#### VAS score

The VAS is a simple method for measuring pain intensity in clinical practice. The basic method is to use a scale with a length of approximately 10 cm, with one side marked with 10 lines [10, 11].

### Statistical analysis

Statistical analysis was performed using SPSS 22.0 (IBM, Armonk, NY, USA). All measurement data (VAS and JOA scores) were expressed by mean ± standard deviation and examined by the Wilcoxon test or Mann–Whitney *U* test. The data were measured by the same deputy chief physician and reviewed by two other deputy chief physicians. The statistical difference level was set at < 0.05.

## Results

### General results

**Fig. 1:**
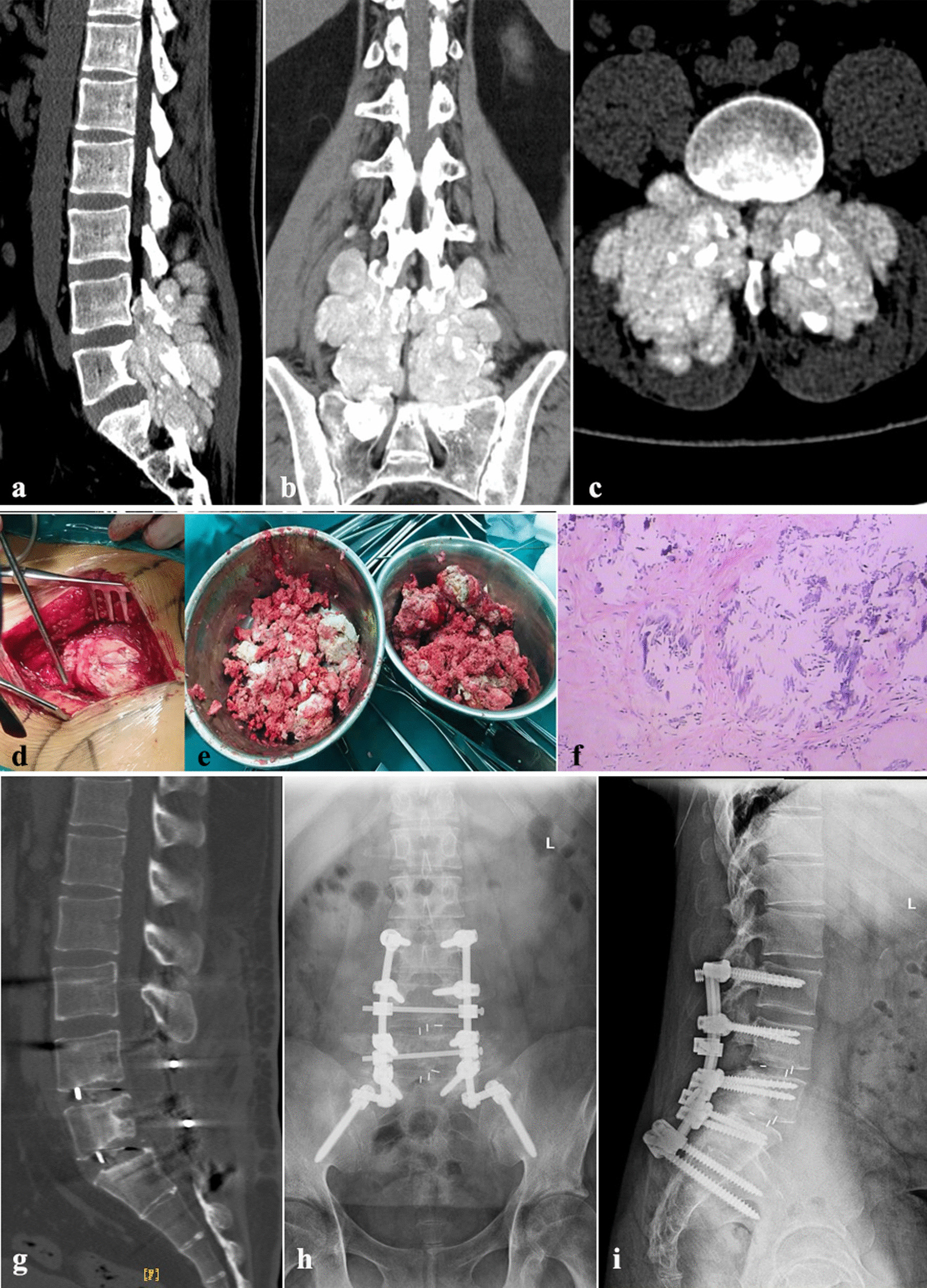
A 26-year-old male patient. **a**, **b** and **c**, preoperative lumbar CT showed extensive gout deposition in the lumbosacral region, destruction of adjacent vertebral bodies and attachments, protruding to the spinal canal, secondary L4–S1 spinal canal stenosis and compression of bilateral nerve roots. **d** and **e**, huge gout was showed during the operation. **f**, postoperative pathology showed spinal gout stone crystallization. **g**, one year after operation, CT showed that there was no gout deposition and no loosening of screws. **h** and **i**, X-ray examination showed no loosening of screws 1 year after operation. There was one screw tail cap loosening

**Fig. 2:**
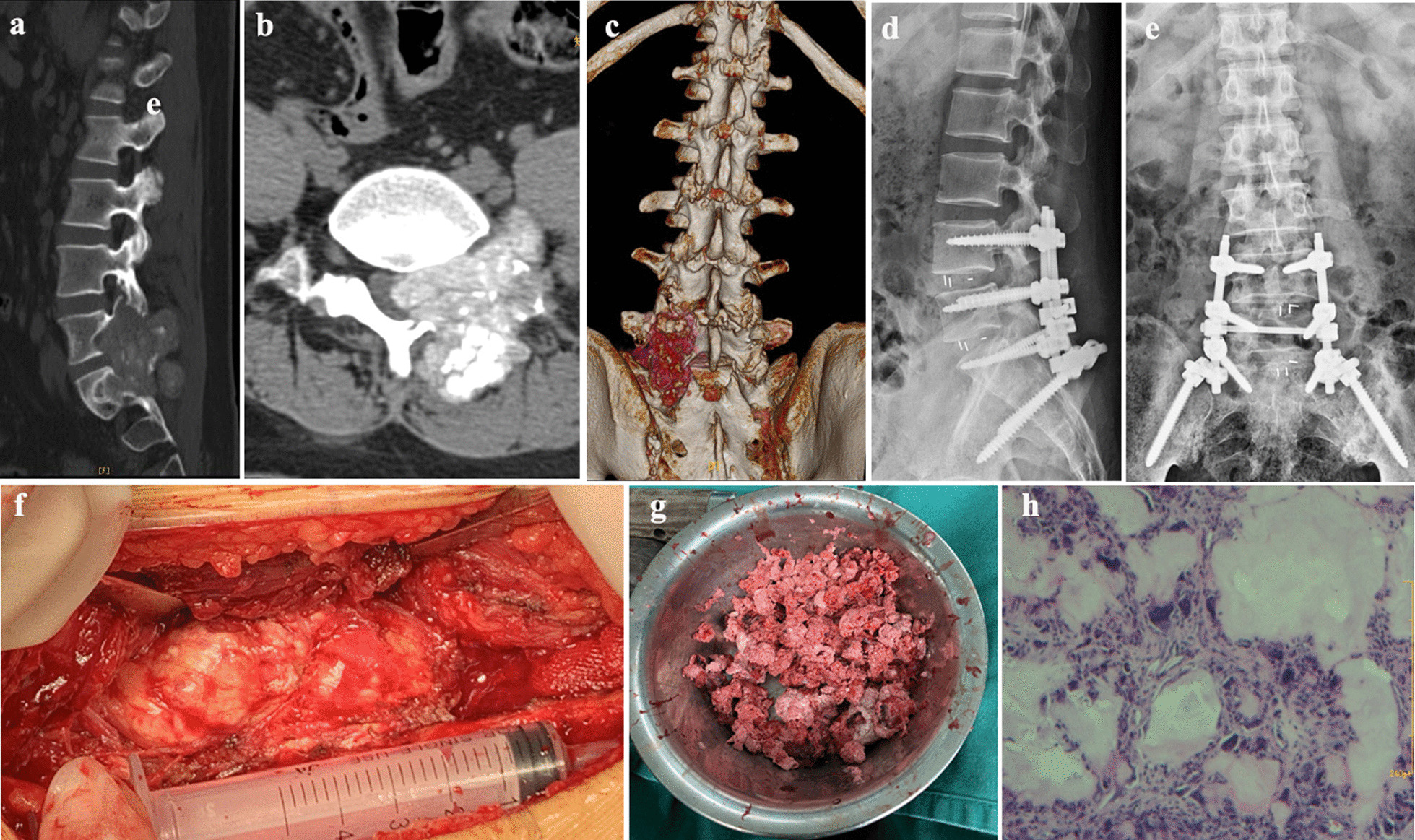
A 39-year-old male patient. **a**, **b** and **c**, preoperative lumbar CT showed extensive gout deposition in the lumbosacral region, destruction of adjacent vertebral bodies and attachments, protruding to the spinal canal. **d** and **e**, one year after operation, X rays showed that there was no gout deposition and no loosening of internal fixation. **f** and **g**, huge gout was showed during the operation. **h**, postoperative pathology showed spinal gout stone crystallization

The general results of the seven patients are shown in Table 1. The seven patients all underwent posterior decompression, fusion and fixation in the treatment of spinal gout. The wound healed well after 1 month follow-up. During follow-up, CT examination of seven patients showed that the internal fixation position was satisfactory and there were no screws loosening. There was only one screw tail cap loosening. In one-year follow-up of 6 cases of lumbar spine, the original signs of spinal cord compression were reduced, and the interbody fusion bone grew well and connected with the upper and lower vertebral bodies (Figs. 1 and 2). In one-year follow-up of the cervical spine, the fracture end of the odontoid process of the axis was more hardened and the gap was narrower than the front, so as to achieve the purpose of fusion and stability (Fig. 2).

### Intraoperative observation

The spinal gout had a clear boundary with the surrounding muscle tissue. It could be seen that the gout stone capsule exists, but the boundary with bone tissue was unclear, which may erode the vertebral lamina, vertebral body and spinal facet joint capsule capsule. The intervertebral disc was mostly well preserved.

### Outcome measures

The JOA and VAS scores are shown in Table 2. The JOA score was 13.43 ± 6.55 and the VAS score was 7.43 ± 1.51 preoperatively. The JOA score was 24.43 ± 3.74 and the VAS score was 0.86 ± 0.90 postoperatively at 12 months after surgery. At 12 months after surgery, the JOA and VAS score showed significant improvements when compared with those before surgery (*P* = 0.002 and *P* = 0.000, respectively).

**Table 2:** JOA and VAS scores of the patients before and after 12 months of operation

Number	JOA		VAS	
	Before	After	Before	After
1	6	24	9	2
2	7	24	9	1
3	21	28	7	0
4	13	17	6	1
5	14	26	8	0
6	23	28	5	2
7	10	24	8	0
*U* value	2.00		0.00	
*P* value	0.004^b^		0.002^b^	

JOA and VAS scores of the patients before and after 12 months of operation are shown in Table 2. a Statistical difference between before and 12 months after operation level was set at < 0.05. b Statistically significant difference was set at < 0.01. JOA: Japanese Orthopaedic Association; VAS: visual analogue scale

### Complications

During follow-up, none of the patients had re-surgery of the gout due to actively and reasonably controlling uric acid. No loosening and displacement of screws were reported. There was only one screw tail cap loosening. In the last follow-up CT of 7 cases, there was no obvious accumulation of gout and surrounding bone destruction, and the segmental instability was significantly improved. There was no progressive aggravation of neurological symptoms during follow-up.

## Discussion

Gout is directly related to hyperuricemia caused by purine metabolic disorder and/or reduced uric acid excretion and belongs to the category of metabolic rheumatism [12]. Gout is caused by urate deposition in tissues. Because urate is not easy to penetrate the blood–brain barrier, gout nodules can be formed in almost all tissues except the central nervous system. However, gout invading spine-related structures is still rare in clinic [13–15]. Although the incidence rate of spinal gout is low, it was found that some gouty patients may even account for 14% [16]. At present, the etiology of gout involving the spine is not clear. Spinal gout has no obvious specificity in clinical symptoms and imaging examination, so it is common to misdiagnose and miss diagnosis in clinic. It is suggested that patients with a history of gout or hyperuricemia should be considered the possibility of spinal gout if they have neck, back and waist pain or discomfort. The seven patients analyzed in our study were all male patients, with an average age of 52.57 ± 20.98 years. Two patients were younger than 30 years old, and five patients had a history of hyperuricemia and gout before operation. It also further shows that middle-aged and elderly men suffering from hyperuricemia and gout are the main objects of spinal gout, which can also be seen in young patients. In the retrospective analysis of cases, there were two patients with systemic multiple gout nodules, neck, chest and lumbosacral were involved at the same time, and a large number of gout deposits were removed from lumbosacral during operation. With the improvement of living standards, more middle-aged and young people may have gout and spinal gout. However, due to the few cases analyzed in our study, the etiology of spinal gout is not clear, so more epidemiological data are needed to further explore.

Due to the absence of gout nodules on the body surface, spinal gout usually does not have the typical clinical manifestations of peripheral gouty arthritis, and its symptoms and imaging manifestations are easy to be confused with tumors, infections and other diseases [17], so there is a lack of effective diagnosis and treatment measures and standards, which brings difficulties to the diagnosis and treatment of spinal gout. Hu et al. found that the sensitivity and specificity of DECT in identifying gout were 91.9% and 85.4%, respectively [18]. DECT, as a noninvasive method, can clearly show urate crystals, which is helpful for the diagnosis and follow-up prevention of spinal gout. In this study, preoperative imaging X-ray films showed 0 cases of spinal gout, CT showed 4 cases of spinal gout, and MRI showed 2 cases. Among them, 3 cases were diagnosed as spinal gout by pathological examination. X-ray films cannot show gout in spinal canal and articular process, which has little diagnostic significance. MRI is effective in the diagnosis of early and active gouty arthritis, but it is not sensitive to calcification and has low specificity, which is easy to be confused with other diseases. Although CT is the most sensitive to the deposition of gout in spinal gout and the presence of spinal gout is often found by CT, it is easy to be misdiagnosed when there is no calcified deposition of gout [19]. Therefore, the diagnosis of spinal gouty arthritis often needs to be combined with various laboratory examinations, imaging data and patient history. The incidence rate of gout is seriously underestimated.

Spinal gout can affect all segments of the spine [7]. In this study, the proportion of lumbar vertebrae and cervical vertebrae were 85.7% (6/7) and 14.3% (1/7), respectively, which also proved that lumbosacral vertebrae was the most common affected segment. Although there is no specificity in the involved location of vertebral body, the spinal gout invaded the vertebral body, ligament and dura mater. However, the intervertebral disc was basically not involved. This may be related to less blood supply of intervertebral disc, which needs further research.

For the treatment of spinal gout, there are conservative treatment and surgical treatment. Conservative treatment includes diet treatment and drug treatment. 35% of patients with spinal gout have a history of gout more than 3 years and have irregular medication treatment [20]. Richette et al. [21] reported that the blood uric acid decreased significantly in five patients with gout after 6 months of treatment with rasburicase, and the gout decreased in two patients. The prevention and treatment of lumbar gout is long-term work and the education for patients should be strengthened. First of all, the patients should understand the mechanism of the disease. And then the methods of controlling of diet, relieving pain and functional exercise, preventing adhesion and stiffness, and reducing blood uric acid should be provided for the patients. If gout invades the lamina and facet joints leading to great bone destruction, the patient's pain symptoms are serious and affect work and rest. Or the gout is located in the spinal canal and compresses the nerve resulting in numbness, weakness and pain of the lower limbs, and conservative treatment is ineffective. Surgical treatment should be considered. When patients with spinal gout are accompanied by obvious spinal canal stenosis, compression of spinal cord and nerve root, resulting in corresponding clinical symptoms, the surgery of decompression is the main treatment method [22]. When spinal gout is formed, surgical resection is the first choice. Through decompression, bone graft fusion and reconstruction of spinal balance, we can reduce the symptoms of patients, restore the function of patients' spine and improve the quality of life. In our study, seven cases of spinal gout treated by posterior approach decompression, fusion and fixation were analyzed retrospectively. Compared with anterior approach and minimally invasive surgery, posterior incision can fully expose the vertebral body and its accessory structures, remove urate crystals, reduce spinal nerve pressure, and fix the upper and lower vertebral bodies of the lesion through bone grafting and fusion of unstable segments to limit the further development of the disease. Due to the high stress of lumbosacral activities, 2 cases were fixed with internal fixation and iliolumbar external fixator to reduce the stress of sacral and lumbar spine. The wounds of 7 cases healed smoothly without infection and liquid exudation. Compared with those after gout resection in four limbs, the incision was sutured layer by layer after spinal gout resection, which can basically achieve no liquid exudation, reduce the probability of infection and facilitate the growth of the skin. This is one of the advantages of the incision approach of spinal gout [23]. Because gout is a systemic metabolic disease, surgical treatment can only remove the gout, expand the spinal canal, alleviate the compression symptoms of spinal cord or nerve root, and cannot completely cure spinal gout. Therefore, postoperative changes in diet and living habits must be combined with anti-gout drug treatment in order to effectively control spinal gout.

After 12 months of follow-up, the average VAS score was 0.86 ± 0.90, and there was basically no pain and discomfort. In the JOA score, the subjective symptoms, clinical signs, limitation of daily activities and bladder function were significantly improved, and the condition tended to be stable. The muscle strength and bladder dysfunction caused by nerve compression in 3 cases basically returned to normal. At the last follow-up, there were no symptoms of pain, discomfort and nerve root compression. Seven patients were satisfied with the effect of surgical treatment. Through X-ray films or CT plain scans, 7 cases of internal fixation are in place. No screws were loose and no instability of cervical atlantoaxial joint was found. There was only one screw tail cap loosening. The fusion and growth of lumbar and lumbosacral vertebrae were satisfied and obvious bone connection could be seen. During the follow-up examination of all patients, there was no obvious recurrence of gout in the surgical segment, no bone erosion in atlantoaxial vertebra, and no compression of spinal canal gout in lumbar vertebra and lumbosacral vertebra.

## Conclusion

Our study has limitations. This is a descriptive and retrospective analysis, with the inclusion of a small sample size due to a single-center study. Posterior decompression, fusion and fixation can effectively reduce the compression of spinal gout at the operative level. In terms of treatment mechanism, it can stabilize the vertebral body, remove gout and directly relieve the compression of spinal cord. It is an effective means to treat spinal gout.
